# Reduction in H3K4me patterns due to aberrant expression of methyltransferases and demethylases in renal cell carcinoma: prognostic and therapeutic implications

**DOI:** 10.1038/s41598-019-44733-y

**Published:** 2019-06-03

**Authors:** Aman Kumar, Niti Kumari, Ujjawal Sharma, Sant Ram, Shrawan Kumar Singh, Nandita Kakkar, Karanvir Kaushal, Rajendra Prasad

**Affiliations:** 1 Department of Biochemistry, Post Graduate Institute of Medical Educational and Research, Chandigarh, India; 2 Department of Urology, Post Graduate Institute of Medical Educational and Research, Chandigarh, India; 3 Department of Histopathology, Post Graduate Institute of Medical Educational and Research, Chandigarh, India

**Keywords:** Renal cell carcinoma, Oncogenes

## Abstract

Renal cell carcinoma (RCC) is the leading cause among cancer-related deaths due to urological cancers, which results in response to combination of genetic and epigenetic factors. Histone methylations have been implicated in renal tumorigenesis but their clinical significance and underlying pathology are unexplored. Here, we elucidated the histone 3 lysine 4 (H3K4) methylation patterns in clear cell RCC and its underlying pathology. Lower cellular levels of H3K4 mono-methylation, -dimethylation and –tri-methylation were fraternized with higher TNM staging and Fuhrman grading as well as tumor metastasis. Further, the expression profile of 20 H3K4 modifiers revealed the significant over-expression of histone demethylases compared to methyltransferases, indicating their role in the reduction of H3K4 methylation levels. In view of above facts, the role of LSD2 and KDM5A demethylases in RCC pathogenesis were explored using respective siRNAs. The RCC cells exhibited reduced cell viability after knockdown of LSD2 and KDM5A genes with concomitant induction of apoptosis. In addition, propidium iodide staining demonstrated an arrest of RCC cells at S-phase and sub-G1 phase of the cell cycle. Taken together, these observations provide new pathological insights behind the alterations of H3K4 methylation patterns in ccRCC with their prognostic and therapeutic implications.

## Introduction

Renal cancer is the most common and fatal among urologic cancers, which contributes about 120,000 deaths each year worldwide^1^. Over the past two decades, the prevalence of kidney cancer has been increased^2^. Renal cell carcinoma (RCC) is the dominant form of kidney cancers, which originate from renal tubular epithelial cells and constitute about 85% to 90% of all cases^3^. RCC is a group of divergent subtypes based on morphologic, genetic and pathological features. Particularly, there are four primary subtypes, viz; clear cell renal cell carcinoma (ccRCC ≥ 75%), papillary RCC (10% of all RCC), chromophobe (5% of all RCC) and renal oncocytoma (3 to 5% of all cases)^4^. The tumor progresses generally asymptomatically and is resistant to conventional chemotherapy and radiotherapy. Radical or partial nephrectomy of the tumor is the mainstay of curative therapy. Despite this, the prognosis is very poor especially with ccRCC, thereby 30% of patients eventually develop metastasis^5^.

Many studies have demonstrated that histone modifications involves in the development and progression of cancers^6^. Histone methylation, which is the most prominent post-translational modification (PTM), can occur at lysine and arginine amino acids at the N-terminus of H3 and H4 by substitution of one, two or three methyl groups. Histone lysine methylation can bring about different transcriptional and biological outcomes based on the degree and site of methylation^7^. Two types of histone modifiers, viz. histone lysine methyltransferases (HMTs) and demethylases (HDMs) are group of enzymes which have indispensable functions in potent modulation of chromatin through histone methylation that influences fundamental nuclear processes^8^. Importantly, a large number of cancer specific alterations in histone modifiers were disclosed by systemic genome-wide studies in number of human cancers^9,10^.

The flavin-dependent demethylase family has emanated as potent drug targets for human cancers. The expression of both LSD1 and LSD2 has been implicated in the pathogenesis of various cancers including breast, lung and hepatocellularcarcinoma^11^. Additionally, LSD2 has been fraternized with various biochemical mechanisms including transcriptional control, chromatin rearrangements, heterochromatin formation, growth factor signaling and somatic cell reprogramming^12–14^.

Unfortunately, we don’t have any early biochemical diagnostic marker for RCC, which is an important contributing factor for poor prognosis of disease. A better conception at the molecular etiology of RCC with special references to the intertwined relationship between various genetic and epigenetic factors is utmost important in the development of newer prognostic markers and therapeutic interventions for ccRCC patients.

In this report, we showed that the histone 3 lysine 4 methylation (H3K4me) patterns decreases with the progression of the tumor to higher stages and grades as well as with tumor metastasis. The underlying pathology leading to the altered H3K4me levels in human cancer is not known. Therefore, we have investigated the expression profile of H3K4 modifiers genes, including 13 histone methyltransferases and 7 histone demethylases, and observed the over-expression of HDMs as compared to HMTs, suggestive of their role in reduction of H3K4me patterns in ccRCC. In addition, functional characterization of LSD2 and KDM5A demethylases revealed their role in inhibiting RCC cells viability with the induction of apoptosis and corresponding arrest of cell cycle. Therefore, our results provide the prognostic value of H3K4me patterns in ccRCC. Further, it was demonstrated that demethylases are involved in reducing the H3K4me levels with LSD2 and KDM5A being the putative therapeutic targets for ccRCC.

## Results

### Global levels of H3K4 methylation are associated with tumor staging and grading in ccRCC

To establish whether H3K4me1, H3K4me2 and H3K4me3 levels are correlating with TNM staging and Fuhrman grading, which are well-established prognostic factors for ccRCC, patients were classified into low stage (stage I and II) and high stage (stage III and IV) based on TNM staging. Likewise, according to Fuhrman grading, patients were also categorized as low grade (grade I and II) and high grade (grade III and IV).

**Figure 1:**
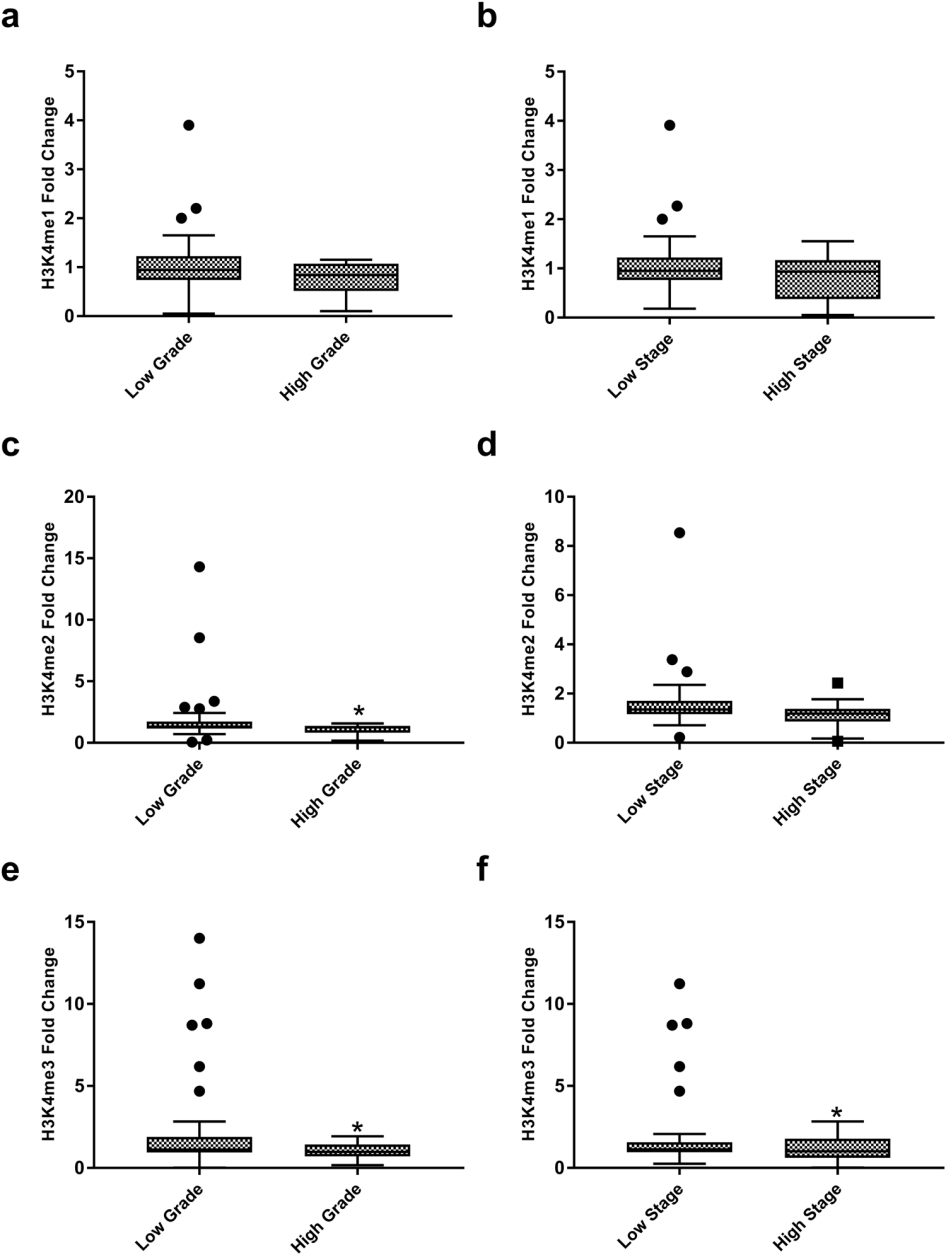
Levels of global histone H3 lysine 4 methylation (H3K4me) in different grades and stages of the tumor. H3K4me levels were measured by ELISA assay. Statistics were calculated using Independent sample t-test. P < 0.05 was considered as significant. (**a–b**) H3K4 mono-methylation (**c–d**) H3K4 di-methylation (**e–f**) H3K4 tri-methylation. (*P < 0.05).

The levels of H3K4me1 were found to be decreasing with both higher TNM stages (p = 0.06) and higher Fuhrman grades (p = 0.092; Fig. 1a,b). Similarly, H3K4me2 levels were also reduced in higher stages (p = 0.096) and higher grade patients (p = 0.045; Fig. 1c,d). A similar trend was also observed in case of H3K4me3; levels were declining with the advancement of the tumor to high stages (p = 0.026) and high grades (p = 0.023; Fig. 1e–f). Although in general, all the three modifications were decreased with progression of cancer to higher stages and grades, a significant reduction was observed in the levels of H3K4me2 with tumor grades and H3K4me3 with stages and grades of ccRCC. Taken all together, these findings are indicative of the role of H3K4me levels in the prognosis of ccRCC.

### Association of global H3K4 methylation levels with tumor metastasis

**Figure 2:**
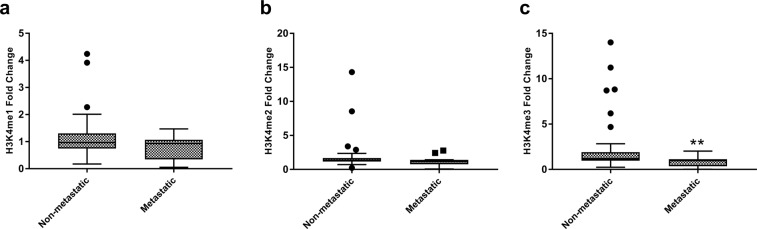
Global histone H3 lysine 4 methylation (H3K4me) levels between metastatic and primary tumors. H3K4me levels were measured by ELISA assay. Independent sample t-test was employed for obtaining statistics. (**a**) H3K4me1 (**b**) H3K4me2 (**c**) H3K4me3. (**P < 0.01).

**Figure 3:**
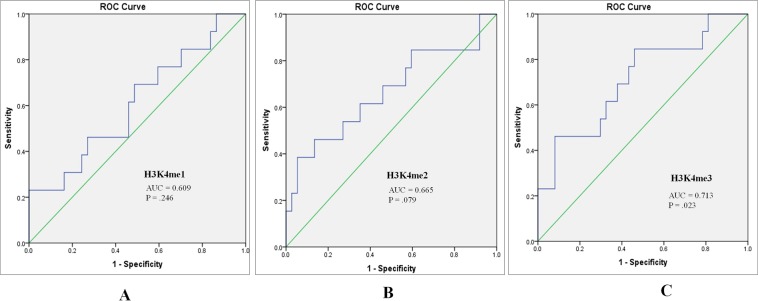
Receiver operator characteristic (ROC) curves assessing the performance of (**A**) H3K4me1 (**B**) H3K4me2 (**C**) H3K4me3 levels as a marker for discrimination between metastatic and non-metastatic tumors (AUC: Area under the curve).

Further, we attempted to establish any correlation between H3K4me1, H3K4me2 and H3K4me3 levels with metastasis of tumors. Remarkably, a reduction in all the three forms of H3K4me levels was observed with metastasis of the tumor. However, this observed reduction in H3K4me3 levels was statistically significant (H3K4me1; p = 0.073, H3K4me2; p = 0.083, H3K4me3; p = 0.006; Fig. 2a–c). Further, the performance of H3K4me1, H3K4me2 and H3K4me3 in predicting metastasis was assessed using Receiver operative characteristics (ROC) curve, which showed that H3K4me3 might be a fair predictor for tumor metastasis with Area under curve (AUC) of 0.713 (p = 0.023; Fig. 3).

### Assessment of HMTs and HDMs gene expression

**Figure 4:**
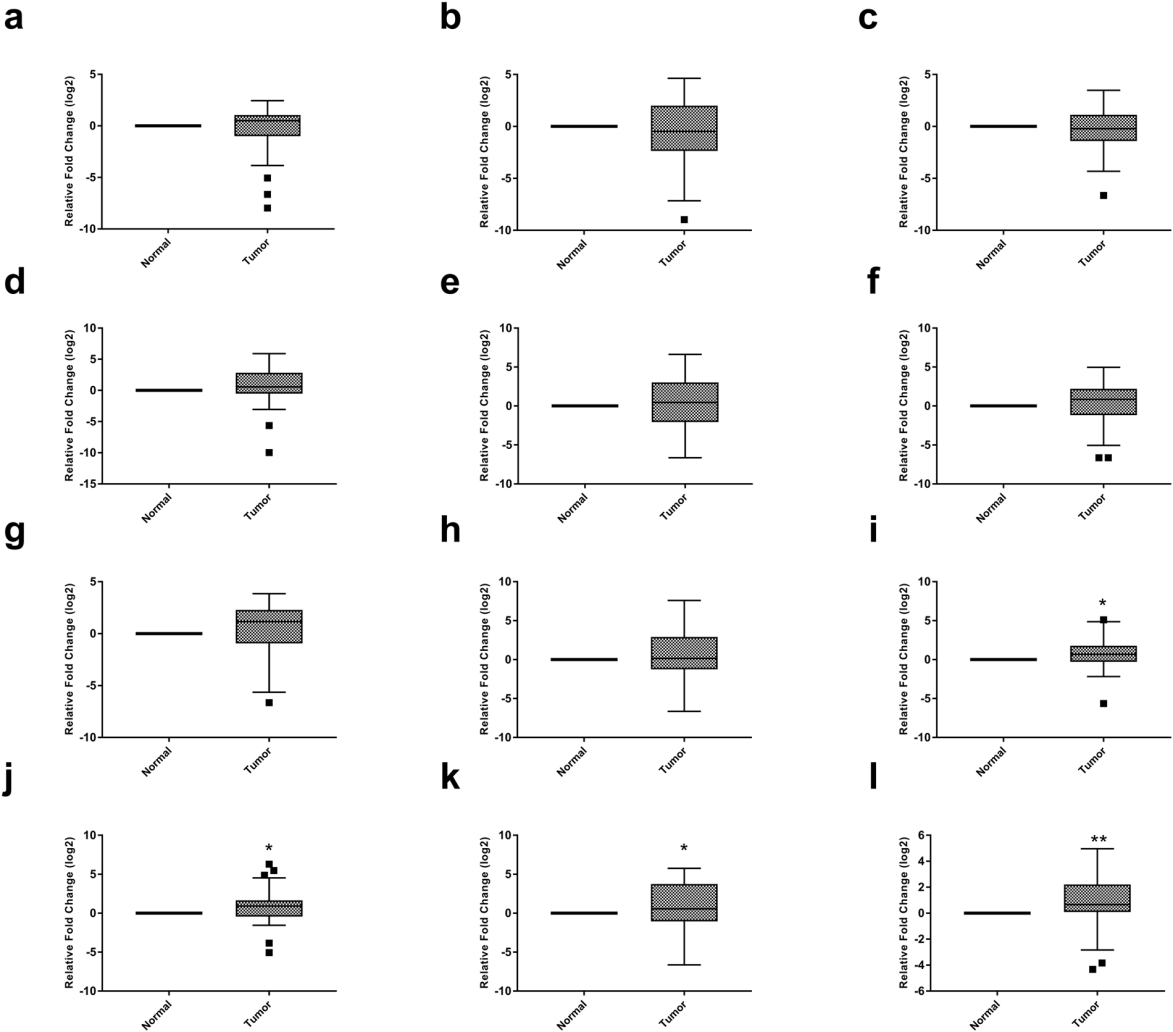
Box plots showing expression profiles of H3K4 lysine methyltransferases in normal and tumor renal tissues. Statistical analysis was obtained by one sample t-test (**a**) MLL4 gene (**b**) SMYD3 gene (**c**) ASH2 gene (**d**) MLL3 gene (**e**) MLL5 gene (**f**) KMT2F gene (**g**) KMT2G gene (**h**) NSD3 gene (**i**) MLL1 gene (**j**) MLL2 gene (**k**) SMYD2 gene (**l**) NSD2 gene. (*P < 0.05; **P < 0.01).

Further, we aimed to find out whether the reduction in observed H3K4me levels was due to the aberrant expression of underlying histone modifiers. Henceforth, gene expression of 13 HMTs and 7 HDMs were analyzed in 50 ccRCC patients and adjacent normal tissues using real-time PCR. The data disclosed a differential transcriptomic profile of H3K4 modifiers. Among 13 HMTs, the three genes namely MLL4, SMYD3, and ASH2 were decresed in tumor tissue as compared to normal; however, this reduction was not statistically significant (p = 0.48, 0.187, 0.429 respectively; Fig. 4a–c). On the other hand, the expression of five genes namely, MLL3, MLL5, KMT2F, KMT2G and NSD3 was augmented in ccRCC. However, this increase was not to the level of significance (p = 0.096, 0.297, 0.554, 0.466, 0.155 respectively; Fig. 4d–h). Strikingly, four genes; MLL1 (p = 0.012), MLL2 (p = 0.024), SMYD2 (p = 0.016) and NSD2 (p = 0.004) was significantly increased in ccRCC in comparison to normal tissues (Fig. 4i–l). Surprisingly, the expression of SMYD1 gene was not detected in tumor tissues or adjacent normal renal parenchyma.

**Figure 5:**
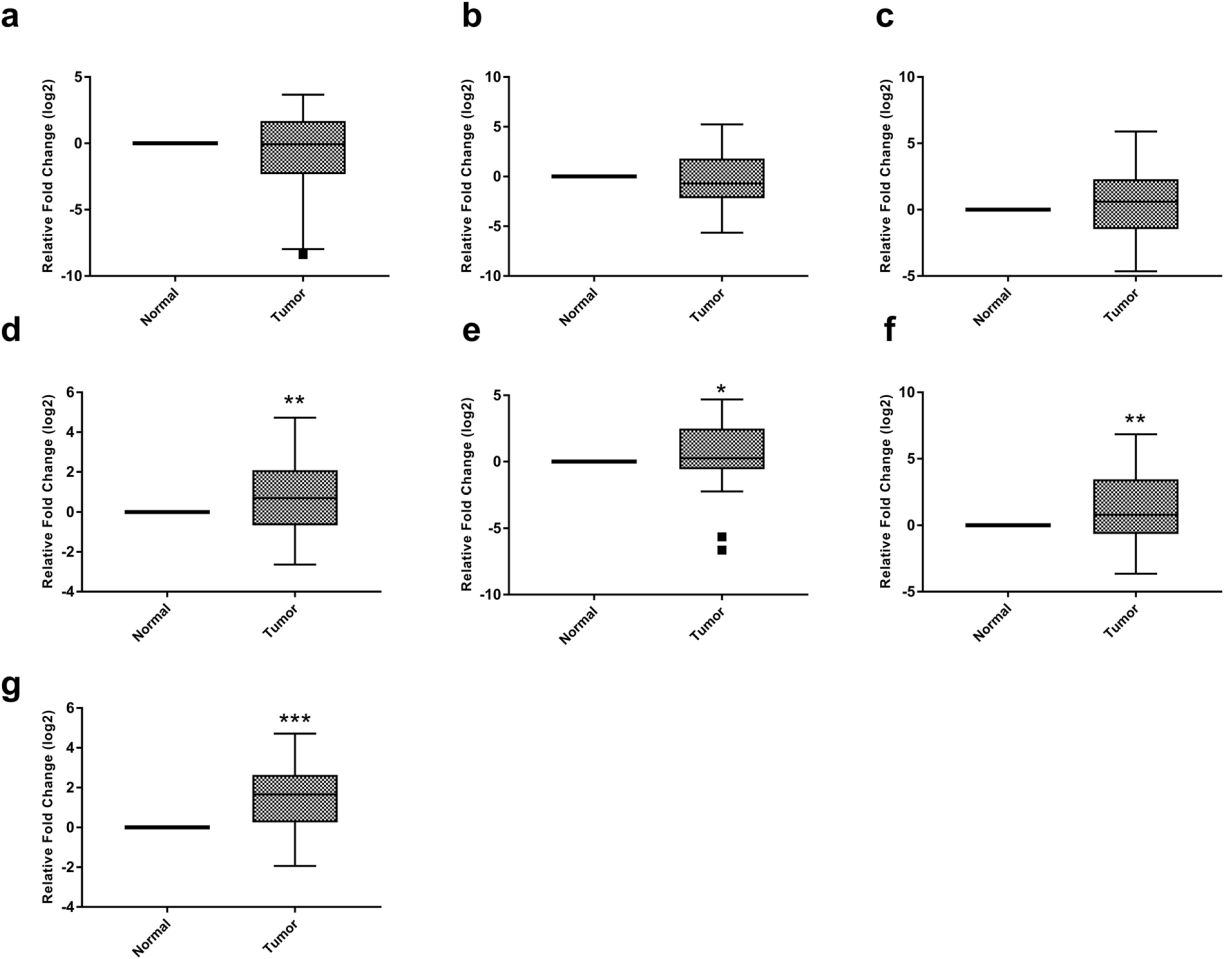
Box plots for expression profile of H3K4 lysine methyltransferases in normal and tumor renal tissues. Statistical analysis was calculated by one sample t-test. (**a**) KDM5D gene (**b**) LSD1 gene (**c**) KDM5C gene (**d**) KDM5A gene (**e**) KDM5B gene (**f**) LSD2 gene (**g**) FBXL10 gene.(*P < 0.05; **P < 0.01; ***P < 0.001).

Out of seven HDMs specific for the H3K4 position, the mRNA levels of KDM5D and LSD1 genes were non-significantly reduced in ccRCC to that of normal tissue (p = 0.166, 0.468 respectively; Fig. 5a,b). Whilst, the expression of KDM5A (p = 0.001), KDM5B (p = 0.047), LSD2 (p = 0.002), FBXL10 (p = 0.0001) and KDM5C genes was augmented in ccRCC and data were statistically significant except KDM5C (p = 0.062; Fig. 5c–g). The clustered heat map for all the 20 genes was created using MeV (Multiple Experiment Viewer; Fig. S2).

**Figure 6:**
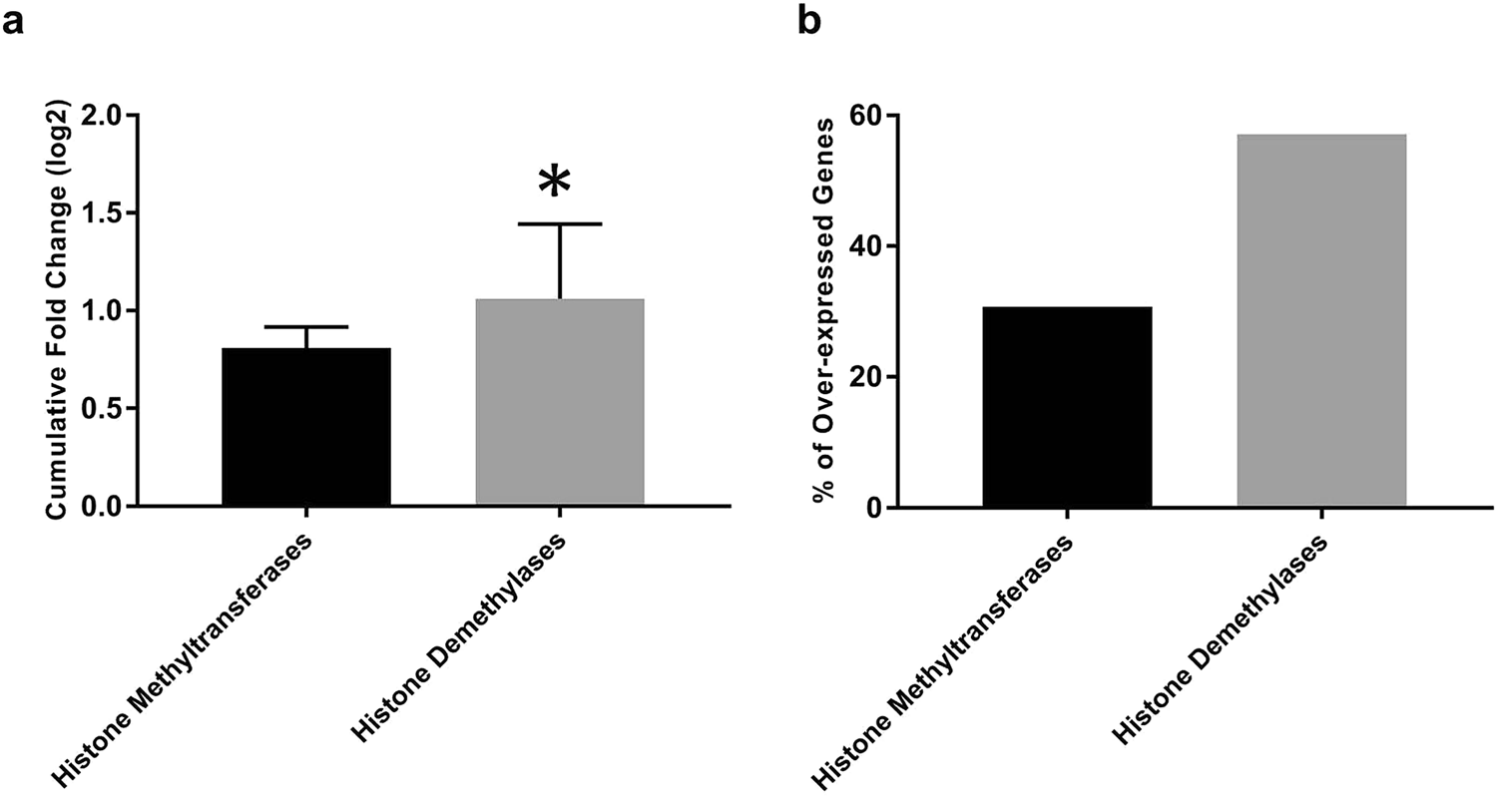
Bar diagram showing a comparison between histone methyltransferases and demethylases. (**a**) Cumulative gene expression (**b**) Percentage of genes significantly over-expressed. Data was analyzed by independent sample t –test (*P < 0.05).

Interestingly, comparative data on HMTs and HDMs genes expression revealed that higher percentage of HDMs were significantly up-regulated with a cumulative expression of about 1.31 times higher as compared to HMTs, indicating overweight of HDMs in ccRCC (p = 0.032; Fig. 6a,b). These findings revealed that up-regulation of HDMs relative to HMTs is involved in the reduction of H3K4me patterns in ccRCC.

### Functional characterization of LSD2 and KDM5A in ccRCC pathogenesis

Due to the involvement of HDMs in reducing the H3K4me levels, we explored their therapeutic potential for ccRCC using two RCC cell lines; A498 and ACHN. For functional characterization, two demethylases were selected; LSD2 and KDM5A. Knockdown of LSD2 and KDM5A with their respective siRNAs resulted in the reduction of 70–80% mRNA levels after 48 hr of transfection (Fig. S3).

**Figure 7:**
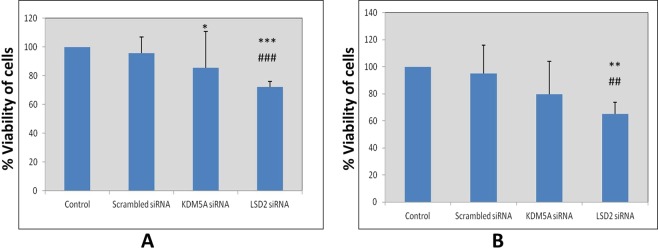
Effect of KDM5A and LSD2 gene silencing on cell viability. (**A**) A498 cells (**B**) ACHN cells. Scrambled siRNA, KDM5A or LSD2 siRNA were used to transfect the cells for 48 hours. MTT assay was used for the assessment of cell viability. Values are expressed in comparison to control cells, which were defined as 100%. Results were analyzed by one-way ANOVA followed by the Bonferroni post hoc correction. (*P < 0.05; **P < 0.01, ***P < 0.001; *in comparison to control, # in comparison to scrambled.

**Figure 8:**
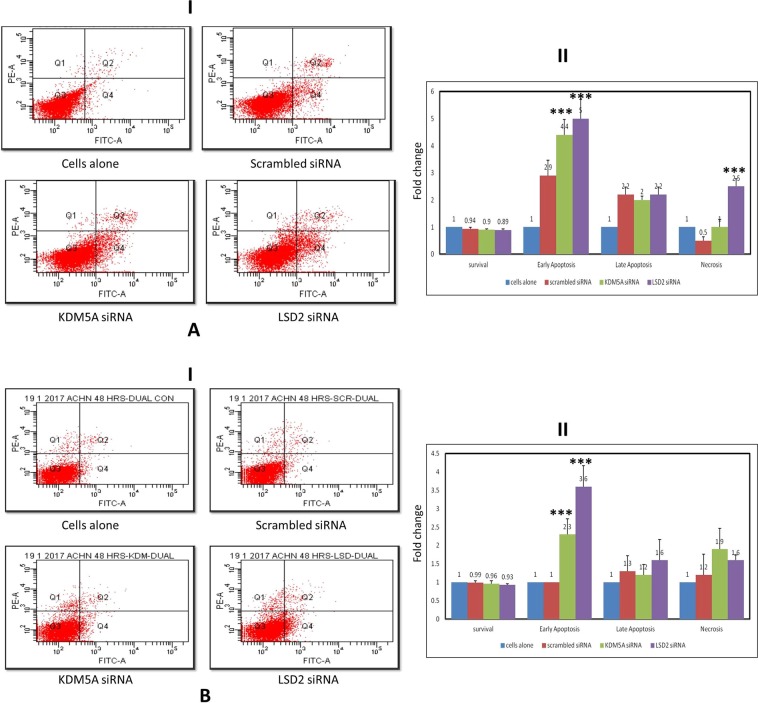
Effect of KDM5A and LSD2 inhibition on cell apoptosis in (**A**) A498 and (**B**) ACHN cell lines. (I) Apoptosis was measured by flow cytometry followed by Annexin V-FITC/PI staining. Data are represented as dot plots. (II) Graphical depiction of flow cytometric data. All values are stated as fold change with mean ± S.D. One-way ANOVA was used for data analysis followed by the Bonferroni post hoc correction. (***P < 0.001).

**Figure 9:**
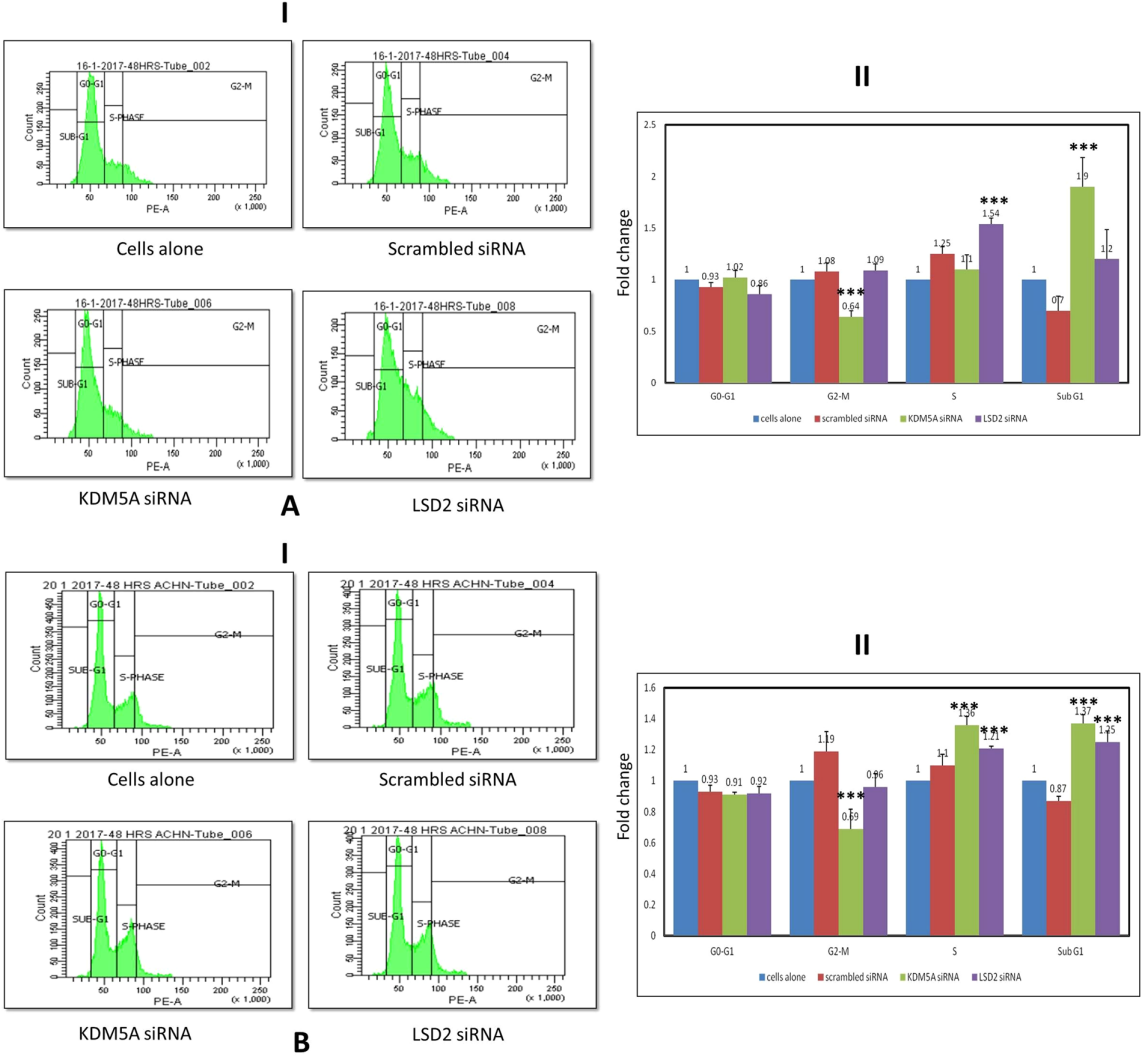
Effect of KDM5A and LSD2 silencing on cell cycle distribution after 48 hours of treatment in (**A**) A498 and (**B**) ACHN cell lines. (I) Cell cycle analysis was carried out by flow cytometry. (II) Graphical illustration of flow cytometry results. Values are represented as the fold change with mean ± S.D. Results were calculated using one-way ANOVA followed by the Bonferroni post hoc correction. (***P < 0.001).

Cell viability was assessed after inhibition of LSD2 (p_A498_ = 0.004; p_ACHN_ = 0.006) and KDM5A (p_A498_ = 0.036; p_ACHN_ = 0.15) levels in A498 and ACHN cell lines. A reduction in cell viability was documented in RCC cell lines. Whereas this reduction was highly significant in case of LSD2 inhibited cells (Fig. 7). Moreover, to investigate whether this reduction in cell viability was due to apoptosis, cell apoptosis was analyzed using flow cytometry. Apoptosis assay revealed induction of early apoptosis in A498 cells after silencing LSD2 (p_A498_ = 0.0001; p_ACHN_ = 0.0001) and KDM5A (p_A498_ = 0.0001; p_ACHN_ = 0.0001) genes (Fig. 8A). Similarly, in ACHN cell line, both LSD2 and KDM5A inhibited groups showed marked accretion in cell apoptosis (Fig. 8B). Further, cell cycle analysis was performed using PI staining which showed the arrest of A498 cells at S and sub-G1 phase in case of LSD2 (p_A498_ = 0.0001; p_ACHN_ = 0.0001) and KDM5A (p_A498_ = 0.0001; p_ACHN_ = 0.0001) knockdown, respectively (Fig. 9A). The ACHN cells were also arrested in S-phase and sub-G1 phase of the cell cycle on silencing of LSD2 (p_A498_ = 0.0001; p_ACHN_ = 0.0001) and KDM5A (p_A498_ = 0.0001; p_ACHN_ = 0.0001) genes (Fig. 9B). In conclusion, these findings revealed that LSD2 and KDM5A silencing inhibited cell proliferation with the induction of apoptosis and corresponding arrest of cell cycle.

## Discussion

Modifications in chromatin architecture conformity because of histone post-translational modifications have been associated with carcinogenesis and turned out to be a potential key regulator of cancer-related pathways^15,16^. These aberrant histone modifications might be because of deregulated expression or activity of major histone modifying enzymes^17^. Therefore, we endeavored to elucidate H3K4 methylation patterns and to characterize alteration in HMTs and HDMs gene expression in ccRCC. Further, attempts were made to exploit them in clinical and pathological implications.

In the present study, an aberrant histone methylation patterns was observed in ccRCC. Histone methylation has been implicated as a good predictor for prognosis in various human cancers^10^. The present study has established a good correlation between the H3K4me marks and currently employed prognostic parameters of ccRCC, which includes TNM staging and Fuhrman grading. Further, to establish their prognostic value, H3K4me levels were compared between metastatic and non-metastatic tumors. All the H3K4me levels were decreasing with metastasis of the tumor, however; data were significant for H3K4me3 only. Moreover, ROC curve also established H3K4me3 to be a fair marker for discrimination of metastatic from non-metastatic tumors. Our data is in accordance with the study that has documented the correlation between levels of H3K4me with higher stages and grades of the tumor and patients survival and recurrence rate, indicating its prognostic implications^18,19^. Similarly, in breast cancer and non-small cell lung carcinoma, reduced levels of H3K4me2 were correlated with poor prognosis^20,21^. In concurrence, our study also supports the concept of global histone modifications as a universal prognostic marker for cancer. However, due to prospective nature of the present study, no correlation could be established between H3K4me patterns and patient’s survival and tumor recurrence.

Further study was carried out to elucidate the underlying pathology behind the altered H3K4me patterns. Several histone modifiers have been implicated in the regulation of the histone methylation on specific positions^9^. Therefore, further study was carried out to study the  gene expression profile of all the twenty H3K4 modifiers, including thirteen HMTs and seven HDMs in ccRCC. The mRNA expression study documented a significant increase in the levels of 4 HMTs viz MLL1, MLL2, SMYD2 and NSD2 in tumor tissues. Till date, the expressions of these modifiers have been studied individually in different cancers. Among 4 members of COMPASS family of H3K4 methylases (MLL1, MLL2, MLL3, and MLL4), MLL1 gene is highly homologous to MLL2 and form identical complexes, which are different from MLL3/4 complexes, which explains the significantly over-expression of only MLL1 and MLL2 genes in our study^10^. Over-expression of SMYD2 and NSD2 was also noticed in various cancers, for instance, esophageal squamous cell carcinoma, pediatric lymphoblastic leukemia, colon and skin cancers^22,23^. Specifically, 2 HMTs; SMYD2 and SETD3 were significantly over-expressed in renal cancers compared to normal and chromophobe RCC compared to oncocytomas^24^.

In the last decade, studies have revealed the prominent role of histone demethylases in the cancer progression^25,26^. Overall, 7 HDMs gene expression levels were evaluated in the current study. Four HDMs, including KDM5A, KDM5B, LSD2, and FBXL10 were significantly up-regulated in ccRCC to that of control. Increased levels of KDM5A, KDM5B and FBXL10 have also been observed in hepatocellular carcinoma, gastric cancer, lung cancer, pancreatic ductal adenocarcinoma, bladder cancer, colorectal cancer and prostate cancer^26,27^. Notably, in-silico studies indicated a significant up-regulation of LSD2 expression in aggressive basal-like breast tumors, indicating an association between over-expression of LSD2 and breast cancer aggressiveness^28^. Notwithstanding, the molecular basis of altered LSD2 expression and its clinical implications in cancer progression are still unclear. It is noteworthy here that LSD2 has been associated with urothelial carcinoma and involved in the regulation of NF-κB. NF-κB was found to be increased in ccRCC due to lack of functional VHL gene, which negatively regulates the NF-κB^29–31^. Henceforth, over-expression of LSD2 gene in the present study might be explained by the augmented levels of NF-κB in ccRCC.

In this study, the cumulative expression of HDMs was 1.31 times higher in comparison to HMTs, indicating the possible role of HDMs in the pathology of ccRCC by observed reduction of H3K4me patterns. According to a recent study, LSD2 was reported as an E3 ubiquitin ligase, which via proteasomal degradation of O-GluNac transferase inhibited the growth of lung cancer cell line. Hence, indicating the role of LSD2 in the inhibition of development of certain cancers^25^. In view of these findings, the study was further carried out to target HDMs so they can be an ideal strategy for therapeutics in ccRCC. For that, the most significantly over-expressed HDMs (LSD2 and KDM5A) were targeted using gene-silencing strategy. mRNA levels of both LSD2 and KDM5A were significantly reduced in RCC cell lines treated with corresponding siRNAs. Consequently, there was a reduction in the viability of A498 and ACHN cells; however, the effect was more prominent in LSD2 inhibited cells, which are consorted with early apoptosis as observed by FACS analysis. Further, cell cycle analysis data documented an arrest of RCC cells at S and sub-G1 phases of cell cycle. Similarly, down-regulation of LSD2 expression by siRNA impedes the growth of various cell lines of breast cancer. In addition, over-expression of LSD2 augmented the colonies in 2D monolayer culture, inferring that LSD2 might participate in promoting the malignancy of breast cancer cells^28^.

Similarly, KDM5A knock-down in medulloblastoma and breast cancer suppressed cell proliferation with the induction of apoptotic genes expression^32,33^. Therefore, induction of apoptosis observed in this study by inhibiting KDM5A might be due to the activation of apoptotic genes. Taken together, these findings are suggestive of the putative therapeutic potential of both LSD2 and KDM5A demethylases with LSD2 as the target of choice for ccRCC.

## Conclusions

This study clearly demonstrated the low levels of global H3K4me are associated with advanced stage and grade of tumor as well as with tumor metastasis, which is an indication of the universal prognostic value of histone modifications. Further, this study revealed the underlying pathology behind H3K4me patterns alteration, which was due to the over-expression of HDMs compared to HMTs. In addition, the atrophic effect of KDM5A and LSD2 inhibition using siRNA on both A498 and ACHN cell growth by inducing early apoptosis with the concomitant arrest of RCC cells at different phases of the cell cycle overlay the first step towards their therapeutic potential in ccRCC.

## Materials and Methods

### Ethical approval and Informed consent

The study was approved by Institute Ethics Committee of Post Graduate Institute of Medical Education and Research with reference number of NK/1597/Ph.D./10916. All the methods were performed according to the ethical standards of the Institute and Informed consent was taken from each patient participating in the study.

### Patients

Total 50 cases of ccRCC who underwent partial or radical nephrectomy at Advanced Urology Centre of Postgraduate Institute of Medical Education and Research, Chandigarh were enrolled in the study. Detailed demographic profiles of all the 50 patients are listed in Table 1. After nephrectomy, tumor tissue and adjacent normal parenchymal samples were taken separately in RNA later for gene expression analysis and in PBS for histone methylation estimation. Histological subtype, tumor staging, and grading were performed by a specialized histopathologist. Hematoxylin and Eosin staining was carried out on every section to confirm tumor and normal renal tissues.

**Table 1:** Clinical-pathological characteristics of clear cell renal cell carcinoma patients.

Patients, n	50
**Sex**, **n**	
Male,	31
Female,	19
**Age**	
Mean ± SD	54 ± 13.2
Range	23–77
**TNM stage (T)**	
T I	20
T II	8
T III	12
T IV	10
**Fuhrman Grade (G)**	
G I	18
G II	24
G III	5
G IV	3

### Histones extraction

The extraction of histone proteins was performed as per the protocol described by Shechter *et al*.^34^. Briefly; 100 mg of each tissue was homogenized in 1 ml of lysis buffer (10 mM Tris-Cl, 1 mM DTT, 1.5 mM MgCl_2,_ 1 mM KCl and protease inhibitor) and subsequently, the homogenate was incubated for 30 minutes on a rotator. Cells were collected by centrifugation at 10,000 g for 10 minutes and resuspended in 0.4 N H_2_SO_4_, followed by overnight incubation on a rotator. Next day, samples were centrifuged at 16,000 g for 10 minutes. In the supernatant, 33% TCA was added drop-by-drop to precipitate the histones. The solution was incubated for 30 minutes followed by centrifugation at 16,000 g for 10 minutes to precipitate the histones. Histone pellet was washed with ice-cold acetone by spinning at 16,000 g for 5 minutes and air-dried. Histones were dissolved in an appropriate volume of double distilled water and stored at −80 °C for subsequent analysis. All the steps were performed at 4 °C. The concentration of histone protein was determined using Bradford’s method and then, resolved on 15% SDS-PAGE (Fig. S1).

### Measurement of global histone methylations

All the three H3K4 methylation patterns i.e. H3K4 mono-, di- and tri-methylations (H3K4me1/H3K4me2/H3K4me3), were quantified and determined in extracted histones using respective colorimetric Global Histone Quantification Kits (Epigentek, Farmingdale, NY, USA) according to the manufacturer’s instruction. 100 ng histone proteins were used for the assay and absorbance was read at 450 nm. For quantification, a graph of OD versus amount of standards was plotted and the delta OD/ng was determined from the slope.

### RNA isolation and reverse transcription qPCR analysis

Total RNA from 50 tumor and adjacent normal tissues as well as from cell lines were extracted by TRIzol^TM^ reagent (Thermo Fisher Scientific, Inc) as per the manufacturer’s instructions. The concentration, purity, and integrity of RNA samples were assessed using a spectrophotometer and gel electrophoresis. 1 μg of total RNA were used for the synthesis of cDNA using iScript^TM^ cDNA synthesis kit (Bio-Rad, USA). For mRNA expression levels, SYBR green master mix was used with 18s rRNA as a reference gene. The PCR reaction was performed on 7500 Fast real-time PCR system (Applied Biosystems, Germany). Primer sequences were synthesized using bioinformatics tools like Primer Blast and Primer3 Plus. The sequences of these primers are listed in Table 2. The data were obtained using comparative threshold cycle (2^−^ΔΔCT) method^35^.

$${\rm{Fold}}\,{\rm{change}}={2}^{-({\rm{Tumor}}{\rm{\Delta }}\mathrm{Ct}-{\rm{Normail}}{\rm{\Delta }}\mathrm{Ct}),{\rm{where}}{\rm{\Delta }}\mathrm{Ct}={\rm{Ct}}(\mathrm{target}\mathrm{gene})-\mathrm{Ct}(\mathrm{18S}\mathrm{rRNA})}{\rm{.}}$$

**Table 2:** Primer sequences for gene expression analysis.

Gene	Forward Primer (5′-3′)	Reverse Primer (5′-3′)
ASH2	CCAGGAGCGGTCGCAAATG	TCCAAGCCACCGCTTACATC
KMT2F	GCATCTAGATCGTCGTGGCG	TCATTTACACTGCGCGAGGC
MLL1	CAGATAAAGTCCAGGAAGCTCG	GTAATTTCGACAGTGCTTGGC
MLL4	AAGGTGAAAGAGCCAGAGCC	ATGGTCAGGTTACGCAGCAA
MLL3	GAAAATGACACAATGTCGAATGC	TCTAGTAGTCCAATCAGGGAATTCTTC
MLL2	TGCTTGGGATAAAGCCTCTTCAGG	CGATGTTGCGCTTACAGAACA
SMYD1	ACGGCTATATGAAGCTCTAC	CTAAGTCCTTAGTGATGGGG
MLL5	CATAACGGGGTTCGGGTGTC	TGACGTTCGCCTCTGGTAAA
NSD2	GACGCACCGCAGTGTTCTAA	GAGGATTTCTGGTGCCTGCT
NSD3	AGAACGTGCTCAGTGGGATATTGG	TGCTTGGGATAAAGCCTCTTCAGG
SMYD2	GCCGGGAGAGGAGGTTTTTA	TGAGCTTCCGGATTTCCACC
SMYD3	TTCAAGTGATGAAAGTTGGC	CCAGTCTCAGATTCTTCATTG
KMT2G	GAGCGAGCTCCAGAACATGA	GTCTCTGTGCTCTGCGACTT
JARID1A	AGACTCAACACATATGGCGG	CTAGCTTCCGTTTCCGTTTCT
JARID1B	GGAACTTTACCAGACTTTACTTGC	AGGCGTCTCTTCAGTTTTCTC
JARID1C	TTCCTTGCTACGCTCTCACTATGA	TCAAATGGGCGTGTGTTACAC
JARID1D	GACAACCATGCAACTTCGAAA	CCCCACGGGAGCATACTTG
FBXL10	TACCAGGGGGACTTCGTG	GACCCTGAGAGCTTCTCTCTGTA
LSD1	GCTCGGGGCTCTTATTCCTA	CCCAAAAACTGGTCTGCAAT
LSD2	CACGGGGGAGGACAAAGAAA	CATCGGGAGGTGTAGCCATT
18s rRNA	AGTCCCTGCCCTTTGTACACA	GATCCGAGGGCCTCACTAAAC

### Cell culture

Two RCC cell line viz. ACHN and A498 were obtained from the National Center for Cell Sciences (NCCS), Pune, India. Cells were maintained at 37 °C with 5% CO_2_ in Minimum essential medium (AL047S Hi-media) supplemented with 2 mM L-Glutamine, 1 mM sodium pyruvate, NEAA, 10% fetal bovine serum and 100 U/ml penicillin, 100 ug/ml streptomycin^36^.

### siRNA transfection studies

For siRNA transfection studies, cells were allowed to grow at 60–80% confluency and transfected with 100 μM each of scrambled siRNA (EHUEGFP, esiRNAs, Sigma Aldrich) and siRNAs specifically targeting KDM5A (EHU0112051, esiRNAs, Sigma Aldrich) and LSD2 (EHU052581, esiRNAs, Sigma Aldrich) using 5 μl of lipofectamine 2000 (Invitrogen). After 6 hours, the culture medium was replaced with fresh medium to reduce the lipofectamine-mediated toxicity. Transfections were performed as per the manufacturer’s instructions in 6-well and 96-well plate. The cells were then cultured for 48 hours. After that, total RNA was isolated using TRIzol reagent and LSD2 and KDM5A mRNA levels were determined using real-time PCR.

### Cell viability assay

Cell viability was checked using MTT assay as described previously^37^. Briefly, in 96-well plates, 5000 cells/well were seeded and incubated overnight. At 60–80% confluency, cells were treated with respective siRNAs and further cultured for 48 hours. To each well of cells, 10 μl of 3-[4,5-dimethylthiazol-2-yl]-2,5-diphenyltetrazolium bromide (MTT; Sigma-Aldrich) was added. Followed by incubation at 37 °C for 4 hours, DMSO was added and cells were further incubated for additional 30 minutes at room temperature. Absorbance was measured at a wavelength of 570 nm with a reference wavelength of 630 nm.

### Cell apoptosis assay using annexin V and propidium iodide

Annexin V–FITC and propidium iodide (PI) double staining (Annexin V-FITC Apoptosis Detection Kit, APOAF, Sigma Aldrich) was used to measure cell apoptosis by flow cytometry as per manufacturer’s instructions. About 1 × 10^6^ cells were harvested after 48 hours of respective siRNAs treatment. The cells were resuspended in the annexin-binding buffer after washing with PBS. Thereafter, 0.5 μl annexin V–FITC and 1 μl PI was added to the cells. The solution was incubated for 10 minutes at room temperature away from light, which was followed by flow-cytometric analysis (BD FACS Canto II). The data are represented as dot plots, annexin V–FITC versus PI.

### Cell cycle analysis

Cells were collected after 48 hours of treatment with their respective siRNAs and fixed in 70% ethanol for overnight at −20 °C. Next day, the cell pellet was made by centrifugation and resuspended in citrate buffer (Sodium citrate, Triton-X, RNase). The solution was incubated for 15 minutes at 4 °C. Then, 5 μg of PI was added to each tube containing cells and were subjected to flow cytometry (BD FACS Canto II) for analysis^36^.

### Statistical analysis

Statistical analysis was done using SPSS software (v.20.0; SPSS Inc., Chicago, IL). Independent sample t-test was used to analyze the relationship between the clinicopathological features and global H3K4 methylation levels. The change in gene expression between tumor and normal tissues was calculated by one samplet-test. Comparison of more than two variables was performed by one-way ANOVA followed by the Bonferroni post hoc correction. ROC curve was plotted to assess the prognostication of H3K4 methylation and AUC was calculated. A P-value of < 0.05 was considered as significant.

## Supplementary information

Reduction in H3K4me patterns due to aberrant expression of methyltransferases and demethylases in renal cell carcinoma: prognostic and therapeutic implications.

Dataset 1

## Data Availability

Data are included in the supplementary information files.
