# Supplementary material for: Reduction in H3K4me patterns due to aberrant expression of methyltransferases and demethylases in renal cell carcinoma: prognostic and therapeutic implications
Source: Sci Rep. 2019 Jun 3;9:8189. doi: 10.1038/s41598-019-44733-y (PMC6546756; doi:10.1038/s41598-019-44733-y)
Supplement: Supplementary file 1 — Reduction in H3K4me patterns due to aberrant expression of methyltransferases and demethylases in renal cell carcinoma: prognostic and therapeutic implications. [file 41598_2019_44733_MOESM1_ESM.pdf]

Title: Reduction in H3K4me patterns due to aberrant expression of methyltransferases and demethylases in renal cell carcinoma: prognostic and therapeutic implications.

Aman Kumar[1], Niti Kumari[1], Ujjawal Sharma[1], Sant Ram[1], Shrawan Kumar Singh[2], Nandita Kakkar[3], Karanvir Kaushal[1], Rajendra Prasad[1*]

[1]Department of Biochemistry, [2]Department of Urology, [3]Department of Histopathology, Postgraduate of Institute of Medical Education and Research, Chandigarh, India

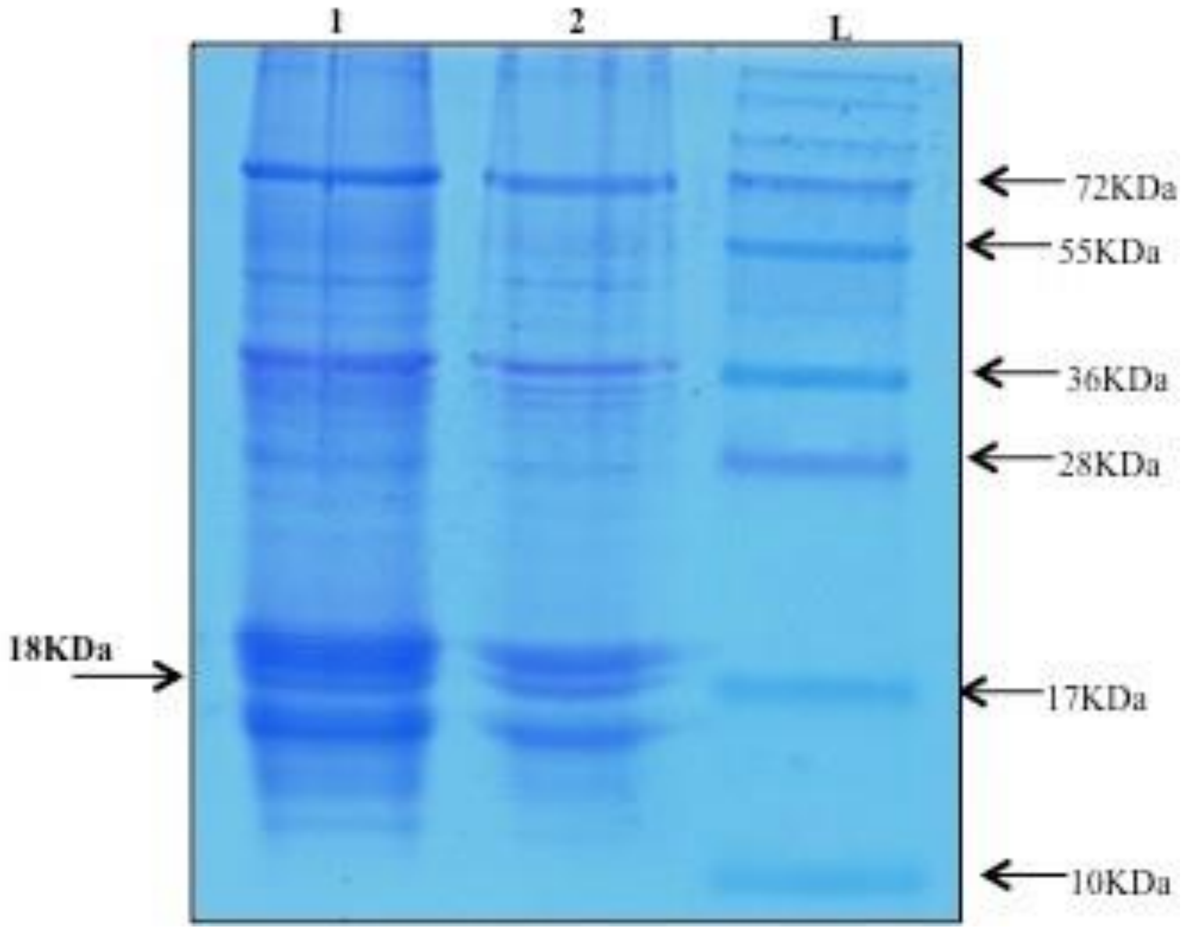

Figure S1: SDS-PAGE of isolated histone proteins. Acid-extracted histone proteins were run on 15% SDS-PAGE. 10 µg and 5 µg of proteins were loaded into the lane 1 and 2 respectively and L denotes the known protein marker. The band around 18 KDa indicates the histone H3.

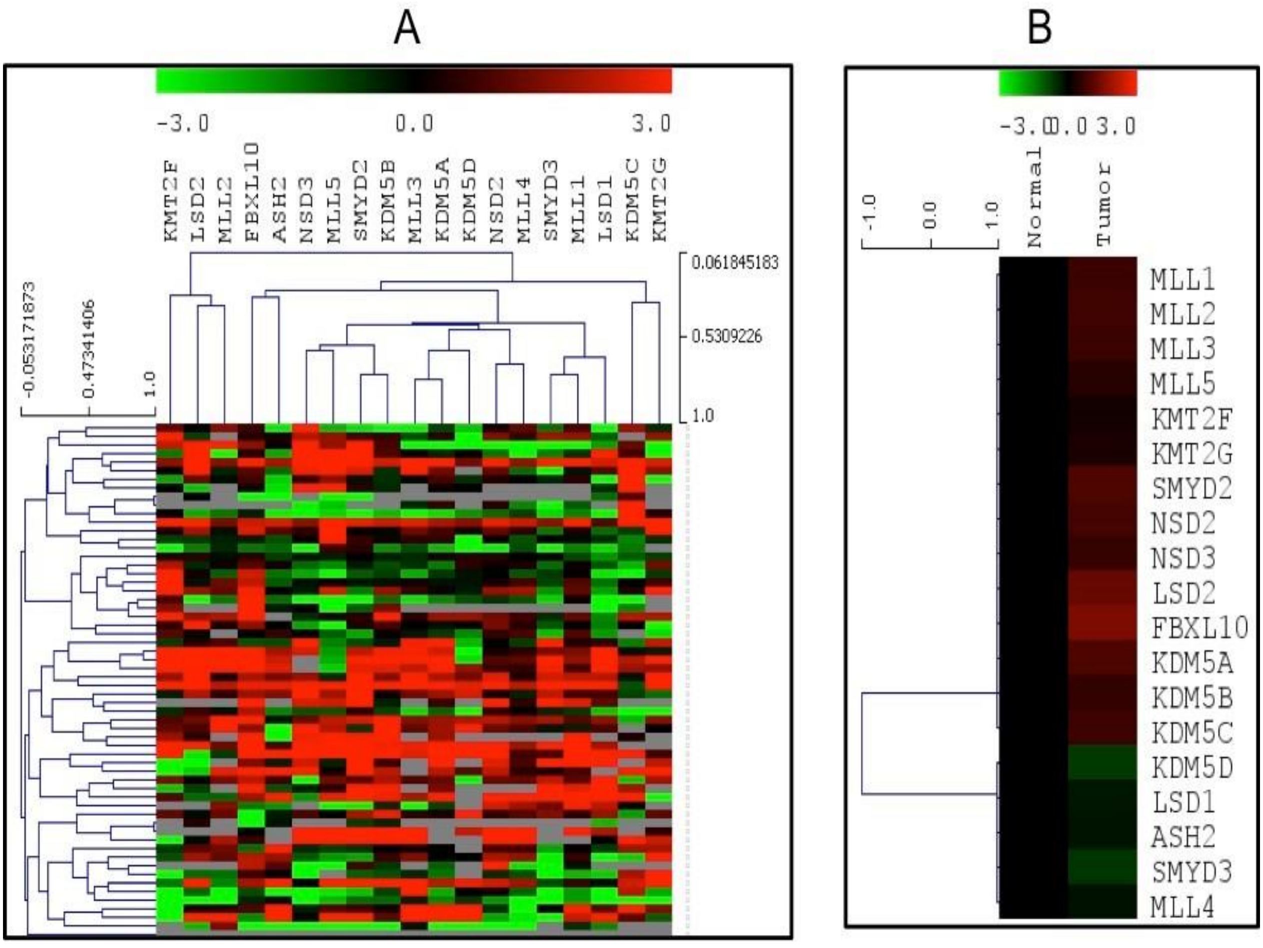

Figure S2: Clustered heat map A) showing the expression of 20 H3K4 modifiers in all the 50 ccRCC patients B) the results presented as a mean value of each gene.

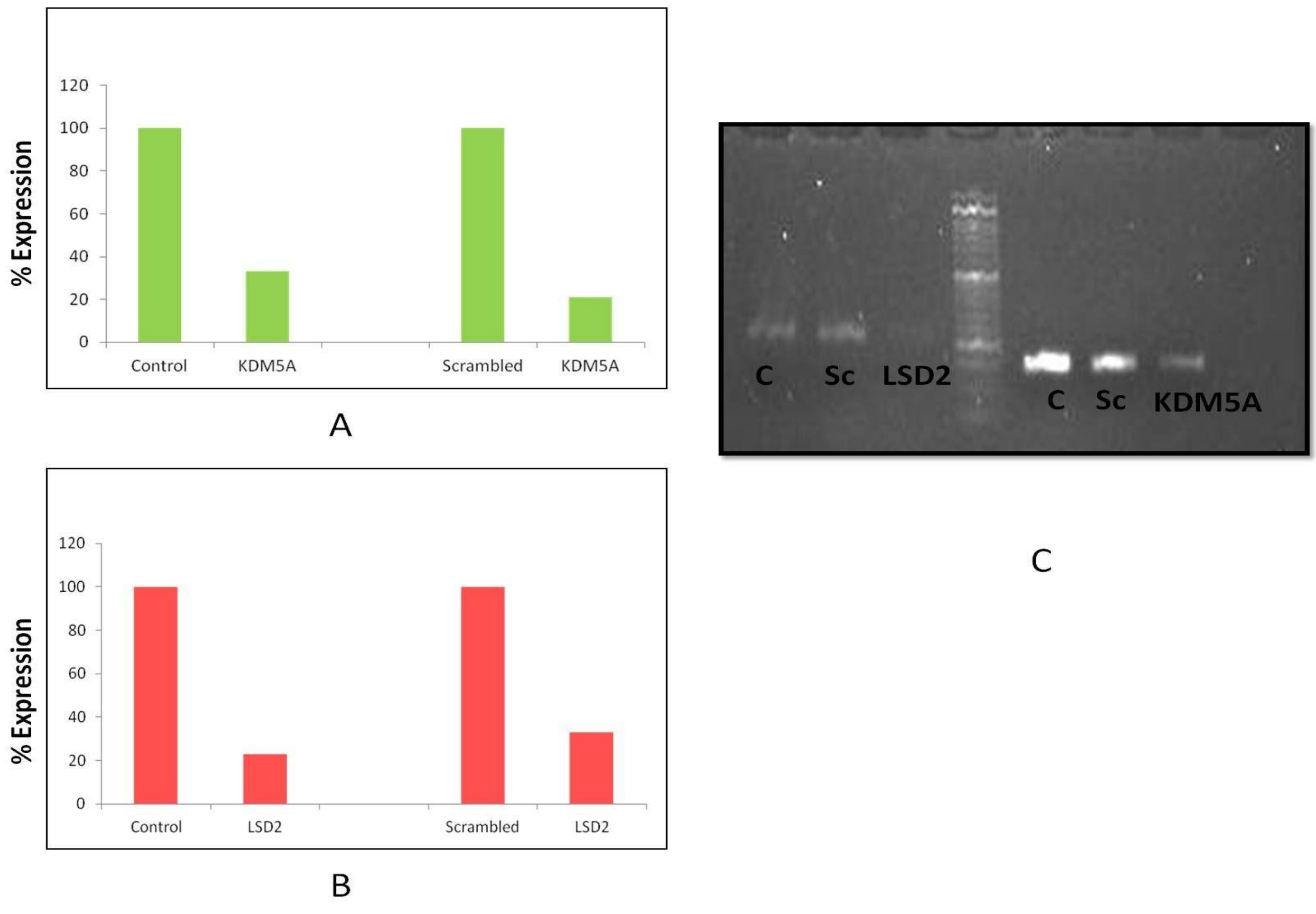

Figure S3: Real-time PCR analysis of mRNA levels after 48 hours of siRNA treatment as compared to control and scrambled group. A) KDM5A gene B) LSD2 gene. C) 2% agarose gel of amplified products. L denotes 50 bp ladder. 18sRNA was used as an internal control.
