# Supplementary material for: Reduction in H3K4me patterns due to aberrant expression of methyltransferases and demethylases in renal cell carcinoma: prognostic and therapeutic implications
Source: Sci Rep. 2019 Jun 3;9:8189. doi: 10.1038/s41598-019-44733-y (PMC6546756; doi:10.1038/s41598-019-44733-y)
Supplement: Supplementary file 2 — Dataset 1 [file 41598_2019_44733_MOESM2_ESM.pdf]

Reduction in H3K4me patterns due to aberrant expression of methyltransferases and demethylases in renal cell carcinoma: prognostic and therapeutic implications.

Aman Kumar[1], Niti Kumari[1], Ujjawal Sharma[1], Sant Ram[1], Shrawan Kumar Singh[2], Nandita Kakkar[3], Karanvir Kaushal[1], Rajendra Prasad[1*]

[1]Department of Biochemistry, [2]Department of Urology, [3]Department of Histopathology, Postgraduate of Institute of Medical Education and Research, Chandigarh, India

Gene expression data:

S. No.	Name of Gene	Fold Change (log_2) in tumor with respect to normal Mean±SEM	p-value
**1.**	ASH2	-0.2352 ± 0.2962	0.429
**2.**	KMT2F	0.2455 ± 0.4100	0.554
**3.**	MLL1	0.7038 ± 0.2689	0.012
**4.**	MLL4	-0.2186 ± 0.3080	0.48
**5.**	MLL3	0.7077 ± 0.4161	0.096
**6.**	MLL2	0.7784 ± 0.3310	0.024
**7.**	SMYD1	**N.D**	-
**8.**	MLL5	0.4764 ± 0.4520	0.297
**9.**	NSD2	0.8268 ± 0.2695	0.004
**10.**	NSD3	0.6571 ± 0.4545	0.155
**11.**	SMYD2	0.9513 ± 0.3828	0.016
**12.**	SMYD3	-0.6145 ± 0.4592	0.187
**13.**	KMT2G	0.3222 ± 0.4358	0.466
**14.**	KDM5A	0.9448 ± 0.2776	0.001
**15.**	KDM5B	0.5966 ± 0.2931	0.047
**16.**	KDM5C	0.7333 ± 0.3821	0.062
**17.**	KDM5D	-0.6895 ± 0.4890	0.166
**18.**	FBXL10	1.482 ± 0.2586	0.0001
**19.**	LSD1	-0.2712 ± 0.3724	0.468
**20.**	LSD2	1.239 ± 0.3818	0.002

N.D . : Not Detectable

Methylation data:

		H3K4me1 (Mean±SEM)	H3K4me2 (Mean±SEM)	H3K4me3 (Mean±SEM)
**Grade**	**Low**	1.002±0.1001	1.883±0.3522	2.212±0.4611
	**High**	0.7675±0.1257	1.086±0.1597	1.041±0.1918
	**p- value**	0.092	0.045	0.023
**Stage**	**Low**	1.092±0.1343	1.713±0.2791	2.309±0.5403
	**High**	0.8291±0.09980	1.152±0.1155	1.113±0.1614
	**p- value**	0.06	0.096	0.026
**Metastasis**	**Low**	1.132±0.1353	1.962±0.3925	2.428±0.5111
	**High**	0.7862±0.1291	1.174±0.2121	0.8777±0.1733
	**p- value**	0.073	0.083	0.006
